# WEENet: An Intelligent System for Diagnosing COVID-19 and Lung Cancer in IoMT Environments

**DOI:** 10.3389/fonc.2021.811355

**Published:** 2022-02-02

**Authors:** Khan Muhammad, Hayat Ullah, Zulfiqar Ahmad Khan, Abdul Khader Jilani Saudagar, Abdullah AlTameem, Mohammed AlKhathami, Muhammad Badruddin Khan, Mozaherul Hoque Abul Hasanat, Khalid Mahmood Malik, Mohammad Hijji, Muhammad Sajjad

**Affiliations:** ^1^ Visual Analytics for Knowledge Laboratory (VIS2KNOW Lab), School of Convergence, College of Computing and Informatics, Sungkyunkwan University, Seoul, South Korea; ^2^ Department of Software, Sejong University, Seoul, South Korea; ^3^ Information Systems Department, College of Computer and Information Sciences, Imam Mohammad Ibn Saud Islamic University (IMSIU), Riyadh, Saudi Arabia; ^4^ Department of Computer Science & Engineering, Oakland University, Rochester, MI, United States; ^5^ Faculty of Computers & Information Technology, Computer Science Department, University of Tabuk, Tabuk, Saudi Arabia; ^6^ Digital Image Processing Laboratory, Islamia College Peshawar, Peshawar, Pakistan; ^7^ Color and Visual Computing Lab, Department of Computer Science, Norwegian University of Science and Technology (NTNU), Gjøvik, Norway

**Keywords:** medical imaging, COVID-19 diagnosis, machine learning, Internet of Medical Things, deep learning, x-ray imaging, cancer categorization

## Abstract

The coronavirus disease 2019 (COVID-19) pandemic has caused a major outbreak around the world with severe impact on health, human lives, and economy globally. One of the crucial steps in fighting COVID-19 is the ability to detect infected patients at early stages and put them under special care. Detecting COVID-19 from radiography images using computational medical imaging method is one of the fastest ways to diagnose the patients. However, early detection with significant results is a major challenge, given the limited available medical imaging data and conflicting performance metrics. Therefore, this work aims to develop a novel deep learning-based computationally efficient medical imaging framework for effective modeling and early diagnosis of COVID-19 from chest x-ray and computed tomography images. The proposed work presents “WEENet” by exploiting efficient convolutional neural network to extract high-level features, followed by classification mechanisms for COVID-19 diagnosis in medical image data. The performance of our method is evaluated on three benchmark medical chest x-ray and computed tomography image datasets using eight evaluation metrics including a novel strategy of cross-corpse evaluation as well as robustness evaluation, and the results are surpassing state-of-the-art methods. The outcome of this work can assist the epidemiologists and healthcare authorities in analyzing the infected medical chest x-ray and computed tomography images, management of the COVID-19 pandemic, bridging the early diagnosis, and treatment gap for Internet of Medical Things environments.

## 1 Introduction

In the beginning of December 2019, a novel infectious acute disease called coronavirus disease 2019 (COVID-19) caused by severe acute respiratory syndrome coronavirus 2 (SARS-CoV-2) has emerged and caused severe impact on health, human lives, and global economy. This COVID-19 disease originated in Wuhan city of China and then spread in several other countries and become a global pandemic (1). This virus is easily transmitted between two persons through petite drops caused by coughing, sneezing, and talking during close contact. The infected person usually has certain symptoms after 7 days that include high fever, continuous cough, shortness of breath, and taste loss. According to the statistical report of the World Health Organization (WHO) (2), COVID-19 affected around 192 countries with 199 million confirmed active cases and 4.2 million confirmed deaths till August 4, 2021. Considering the fast spread and its high contiguous nature, it is essential to diagnose COVID-19 at an early stage to greatly prevent the outbreak by isolating the infected persons, thereby minimizing the possibilities of infection to healthy people. Till date, the most common and convenient technique for diagnosing COVID-19 is the reverse transcription polymerase chain reaction (RT-PCR). However, this technique has very low precision, high delay, and low sensitivity, making it less effective in preventing the spread of COVID-19 (3).

Besides the RT-PCR testing system, there are several other medical imaging-based COVID-19 diagnosing methods such as computed tomography (CT) (4–6) and chest radiography (x-ray) (7, 8). Diagnosis of COVID-19 is typically associated with both the symptoms of pneumonia and medical chest x-ray tests (9, 10). Chest x-ray is the first medical imaging-based technique that plays an important role in the diagnosis and detection of COVID-19 disease. Some attempts have been made in the literature to detect COVID-19 from medical chest x-ray images using machine learning and deep learning approaches (11, 12). For instance, Narin et al. (13) evaluated the performance of five pretrained Convolutional Neural Network (CNN)-based models for the detection of coronavirus pneumonia-infected patients using medical chest x-ray images. Ismail et al. (14) utilized deep feature extraction and fine-tuning of the pretrained CNNs to classify COVID-19 and normal (healthy) chest x-ray images. Tang et al. (15) used chest x-ray images with effective screening for the detection of COVID-19 cases. Furthermore, Jain et al. (16) used transfer learning for the COVID-19 detection using medical chest x-ray images, and they compared the performance of medical imaging-based COVID-19 detection methods.

More recently, several other deep learning-based approaches (17) are presented to overcome the limitations of previous imaging-based COVID-19 detection methods. For instance, Minaee et al. (18) proposed a transfer learning strategy to improve the COVID-19 recognition rate in medical chest x-ray images. They investigated different pretrained CNN architectures on their newly prepared COVID-19 x-ray image datasets and claimed reasonable results. However, their newly created dataset is not balanced and has a smaller number of COVID-19p images compared with non-COVID-19 images. Aniello et al. (19) presented ADECO-CNN to classify infected and noninfected patients *via* medical CT images. They compared their CNN architecture with pretrained CNNs including VGG19, GoogleNet, and ResNet50. Yujin et al. (20) suggested a patch-based CNN approach for efficient classification and segmentation of COVID-19 chest x-ray images. They first preprocessed medical chest x-ray images and then fed them into their proposed network for infected lung area segmentation and classification in medical images. However, their attained performance is relatively low due to the small number of images in their used dataset. Similarly, Yu-Huan et al. (21) presented a joint classification and segmentation framework called JCS for COVID-19 medical chest CT diagnosis. They trained their JCS system on their newly created COVID-19 classification and segmentation dataset. They claimed real-time and explainable diagnosis of COVID-19 in chest CT images with high efficiency in both classification and segmentation. Afshar et al. (22) proposed a deep uncertainty-aware transfer learning framework for COVID-19 detection in medical x-ray and CT images. They first extracted CNN features from images of chest x-ray and CT scan dataset and then evaluated by different machine learning classifiers to classify the input image as COVID or non-COVID.

The current COVID-19 pandemic situation greatly overwhelms the health monitoring systems of even developed countries, leading to an upward trend in the number of deaths on a daily basis. Also, the inaccessibility of healthcare system and required medication to the rural areas caused increase in the loss of human lives. Therefore, an intelligent AI-driven healthcare system is necessarily needed for combating with COVID-19 pandemic and rescuing hospitals and other medical staff. Thanks to the Internet of Things (23–26) and Internet of Medical Things (IoMT) (27, 28) for offering powerful features (i.e., online monitoring, high-speed communication, and remote checkups) that can greatly assist healthcare system of a country against COVID-19 pandemic (29). Also, Healthcare 5.0 with 5G-enabled IoMT environment can effectively improve the accessibility of doctors and nurses to their patients in remote areas, enabling COVID-19 patients to control their health based on daily recommendations from doctors.

Undoubtedly, the deployment of 5G-enabled IoMT protocols can greatly enhance the performance of smart healthcare system by connecting hospitals and patients, transmitting their health-related data between both parties. However, such a smart IoMT healthcare environment demands computationally efficient yet accurate AI algorithms (including both machine learning and deep learning algorithms) (30). Most of the existing deep learning approaches use computationally complex CNN architectures that require high network bandwidth and computational requirements and cannot be employed on resource constrained devices. Thus, the architecture of AI Algorithm (i.e., CNN architecture) to be deployed must meet the requirements of the executional environment (the device used in IoMT environment).

To alleviate the shortcomings of pervious approaches and design energy-efficient model for IoMT-enabled environment, we propose a computationally efficient, yet accurate CNN architecture called WEENet. The proposed architecture is designed to efficiently detect COVID-19 in medical chest x-ray images, requiring limited computational resources. More precisely, the key contributions of this study are summarized as follows:

1. Deep learning-based models require huge amount of medical imaging data to train effectively, but COVID-19 benchmarks have relatively limited number of samples especially for COVID class. To increase the number of images for effective training of the proposed WEENet framework, we applied offline data augmentation techniques on available medical chest x-ray images such as rotation, flipping, zooming, etc. that brought improvements in the performance as evident from the results.
2. WEENet is developed to detect COVID-19 in medical chest x-ray images and support the management of IoMT environments. WEENet uses EfficientNet (31) model as a backbone for feature extraction from chest x-ray images, followed by stacked autoencoding layers to represent the features in more abstract form before the final classification decision.
3. The performance of several deep learning-based models is evaluated using benchmark medical chest x-ray images datasets and eight evaluation metrics including a novel strategy of cross-corpse and robustness evaluation for COVID-19 detection in chest x-ray images. Furthermore, we also compared the performance of our WEENet with other state-of-the-art (SOTA) methods, where it surpassed in terms of several evaluation metrics.

The remainder of the article is organized as follows: ***Section 2*** covers the proposed IoMT-based WEENet framework with a discussion on datasets. In ***Section 3***, we discuss the experimental setups, the experimental results, and their analysis. Finally, ***Section 4*** concludes this paper and suggests future research directions.

## 2 Proposed IoMT-Based WEENet Framework

**Figure 1:**
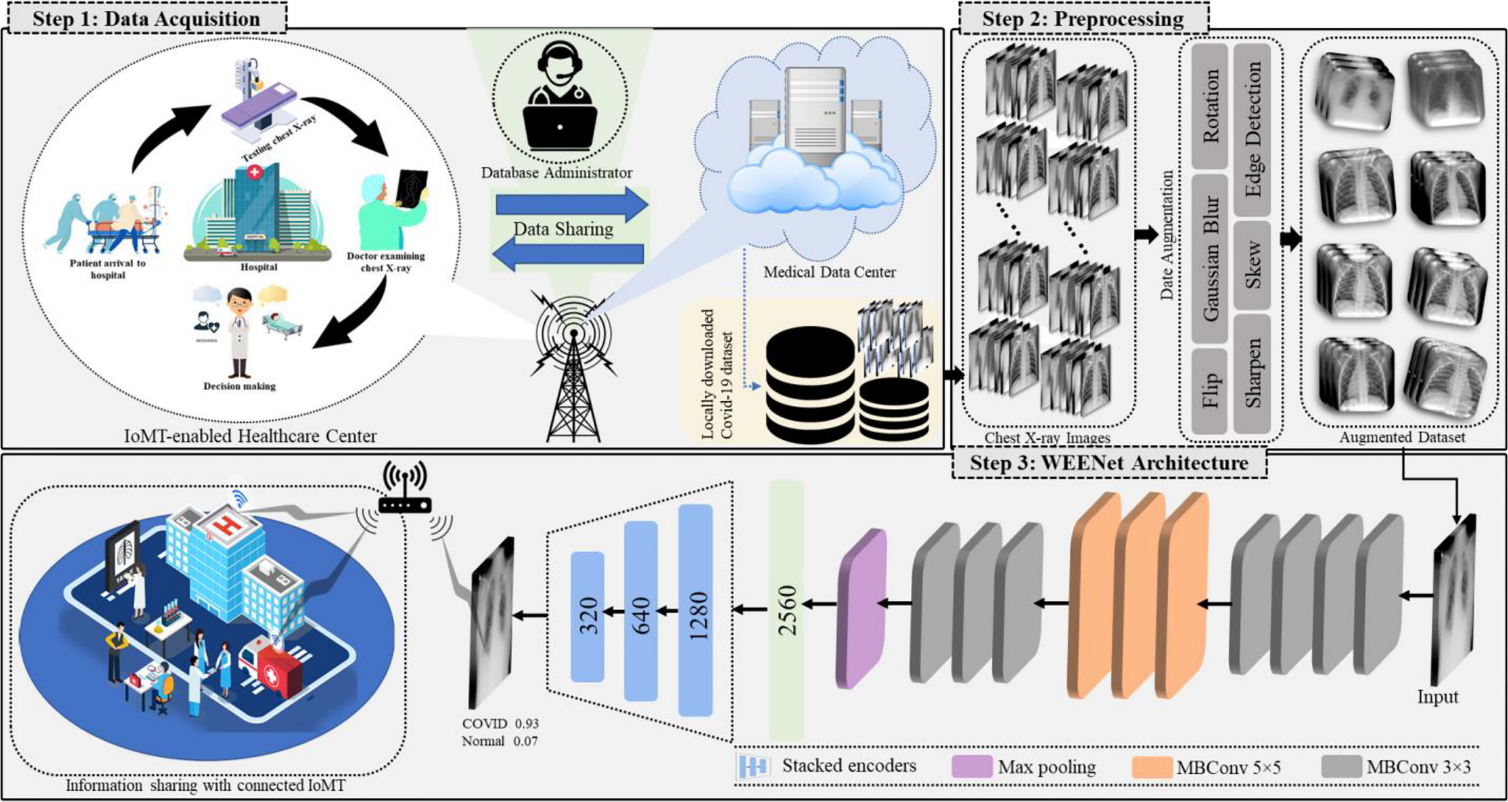
Overview of the proposed WEENet-assisted framework for COVID-19 diagnosis using chest x-ray images with the support of 5G technology and efficient management for IoMT environments.

This section discusses the overall workflow of our WEENet framework in IoMT environment for efficient and timely detection of COVID-19 in x-ray images over edge computing platforms. For better understanding, the proposed WEENET framework is divided into three phases including Data Acquisition, Preprocessing, and WEENet. The first phase presents the detail of data collection from different sources, followed by the second phase which performs extensive data augmentation on the data collected in the first phase to prevent underfitting/overfitting problems. The third phase contains the WEENet architecture which is responsible for COVID-19 detection in x-ray images. The overall graphical overview of our proposed framework with all phases is given in **Figure 1** and explained in the following subsections.

### 2.1 Data Acquisition and Preprocessing

During the pandemic, hospitals around the world produced image data related to COVID-19 (such as medical x-ray and CT images), and some of them are publicly available for research purposes in medical imaging. However, the available COVID-19 image datasets are either not well organized or have lack of balance between positive and negative class samples, which often lead network to model overfitting during the training process. Therefore, the research community is working to organize the available COVID-19 image data and make it usable before utilizing it for early diagnosis of COVID-19. To achieve data diversity and balance between positive and negative class samples, we actively used data augmentation approaches, which not only increase the volume of data but can also significantly improve the classification performance of deep learning models as evident from our experiments.

In this research, we have used three different COVID-19 image datasets namely chest x-ray images (CXI) (18), x-ray dataset COVID-19 (XDC) (32), and COVID-19 radiography database (CRD) (33), where each dataset contains medical chest x-ray images of positive and negative patients. To alleviate the chances of model overfitting and class biasness, we performed extensive data augmentation by equalizing the number of positive and negative class samples in each of the abovementioned datasets. Considering the number of images per class in the dataset, we performed data augmentation with different augmentation ratio for each dataset so that we can obtain balance training data. Following this strategy, we augmented the COVID-19 images of CXI (18) dataset with augmentation ratio of 1:15 such that each image is reproduced in 15 different variants. Similarly, for XDC (32), we used the data augmentation ratio of 1:10 for both positive and negative classes. For CRD (33) dataset, we only augmented the COVID-9 class with the augmentation ratio of 1:3, where each image is reconstructed with its 3 different variants. The proposed data augmentation strategy analyzed different augmentation approaches and then selected the most suitable eight distinct operations on each image of the dataset that include Rotation, Zoom, Width shift, Height shift, Shear, Fill mode, Flip, and Brightness operations before forwarding to our proposed WEENet for training. The details of augmentation operations used in our method are listed in **Table 1**. 

**Table 1:** The operational details of our proposed data augmentation strategy.

No.	Technique	Parameter range
1	Rotation	−25~25
2	Zoom	0.10
3	Width shift	0.01
4	Height shift	0.01
5	Shear	0.1
6	Fill mode	Nearest
7	Flip	Right and left
8	Brightness	0.50, 1.50

It can be noticed that images in original XDC dataset are insufficient for training a deep learning algorithm. Also, the number of positive samples in the CXI dataset is comparatively lesser than negative samples prior to data augmentation process. Similarly, the CRD dataset has also a huge difference between the number of positive and negative samples. On the other hand, the augmented version of the listed datasets can be found well balanced and rich in terms of data diversity, thus more suitable for deep learning-based methods.

### 2.2 EfficientNet: The Backbone Architecture

Several CNN architectures have been explored before choosing the appropriate model that are extensively used in different domain studies such as time series prediction, classification, object detection, and crowed estimation. These architectures include VGG16 (34), VGG19 (34), ResNet18 (35), ResNet50 (35), and ResNet101 (35) that are used by researchers for COVID-19 detection in chest x-ray images, but each CNN model has its own pros and cons. However, researchers investigate these architectures to boost their accuracy by using different scaling strategies to adjust the network depth, width, or resolution. Most of the networks are based on single scaling, that scales only a single dimension from depth, width, and size. Though, scaling two or three dimensions will yield efficiency and suboptimal accuracy. To this end, we investigate EfficientNet that scales all the dimensions through compound scaling technique. This network is developed through leveraging multiobjective architecture search, which optimizes both floating point operations (FLOPs) and accuracy. EfficientNet uses the search space of (36) and ACC (*m*) × [FLOPS (*m*)/*T*]*^w^* as an optimization tool. The ACC (*m*) and FLOPS (*m*) represent the accuracy and FLOPs of model *m* while *T* and *w* are the FLOPs target and hyperparameters, respectively. These terms control the tradeoff between the accuracy and FLOPs. This network comprises several convolutional layers where different-sized kernels are equipped in each layer. The input frame having three channels (R, G, B) corresponds to size such as 224 × 224 × 3. The subsequent layers are scaled down in a resolution that reduces the size of feature maps while the width is scaled up to increase the accuracy. This tool ensures the collection of important features from the input frame. For example, the second layer consists of Width = 112 kernels, and the number of kernels by next convolution is Width = 64. The total maximum kernels used are Depth = 2,560 in the last layer, where the resolution is 7 × 7 which represents the most discriminative features. At the end, we added max pooling layer that is followed by encoding layers and a SoftMax layer for the final classification.

### 2.3 The Proposed WEENet

**Figure 2:**
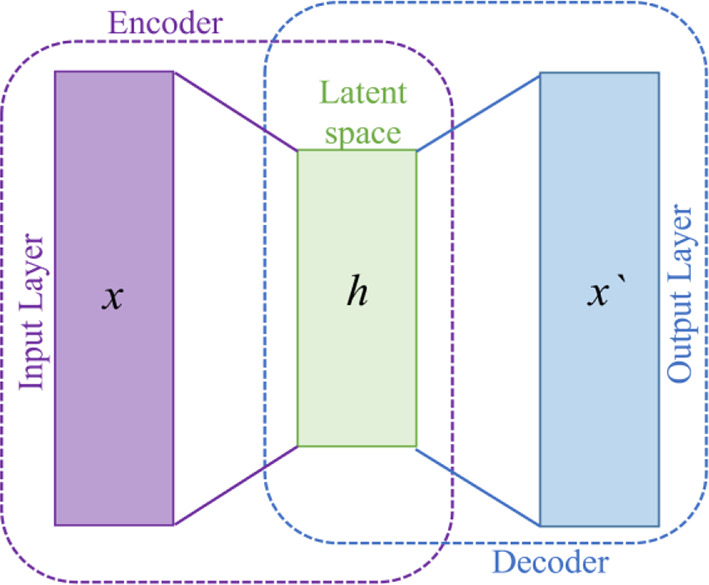
General overview of autoencoder architecture.

The proposed WEENet is based on EfficientNet model followed by encoding layers. EfficientNet is used to extract important features from the input data and then the output is feed forward to stacked encoding layers. The stacked encoding layers are based on autoencoder (37) used to compress the data from high dimension into low dimension, while preserving the salient information from the input data. Autoencoders are a type of deep neural networks that map the data to itself through a process of (nonlinear) dimensionality reduction, followed by dimensionality expansion. The autoencoder models include three layers: input, hidden, and output layer as shown in **Figure 2**. The encoder part is used to map the input data into lower dimension followed by decoding layers to reconstruct it. Let us suppose the input data ${\left(X_{n}\right)}_{n=1}^{N}$ , where *X_n_* belongs to *R^m x 1^, h_n_* is the low-dimension map (hidden state) which is calculated from *X_n_*, and “*O_n_*” is the output decoder. The mathematical representation of encoding and decoding layer is shown in Eq. (1).


(1) $${𝓀}_{n}=F({𝒲}_{1}X_{n}+){𝒷}_{1})$$

here, *F* represents the encoding function, 𝒲_1_ is the weight metrics, and 𝒷_1_ is the bias term. The mathematical representation of the decoding layer is shown in Eq. (2).


(2) $$O_{n}=G({𝒲}_{2}X_{n}+{𝒷}_{2})$$

In this equation, *G* is the decoding function, 𝒲_2_ is the weight metrics, and 𝒷_2_ is the bias term of decoding layer. In our WEENet, we used the encoding part of the autoencoder to represent the features in more abstract form. In these layers, the high-dimension EfficientNet features is encoded to low-dimension features. In the proposed model, two encoding layers are incorporated with EfficientNet architecture. The output of EfficientNet is 2,560 dimension feature vector which is encoded to 1,280 dimension feature vector. Furthermore, 1,280 dimension feature vector is then encoded to 640 dimension feature vector is then 320. The proposed model is trained for 50 epochs, using SGD optimizer with 0.0001 learning rate, and its performance is tested against SOTA as given in ***Section 3***.

## 3 Experimental Results and Discussion

In this section, we evaluated our WEENet on three publicly available COVID-19 datasets and compared the classification performance with other methods. For this, we first provide the details of experimental settings of this research study, followed by information about datasets and metrics for performance evaluation. Subsequently, we compare the proposed WEENet with other SOTA CNN architectures used for COVID-19 classification. Finally, we close this section by emphasizing on the feasibility of our proposed WEENet framework for COVID-19 diagnosis in 5G-enabled IoMT environments.

### 3.1 Implementation Details

**Figure 3:**
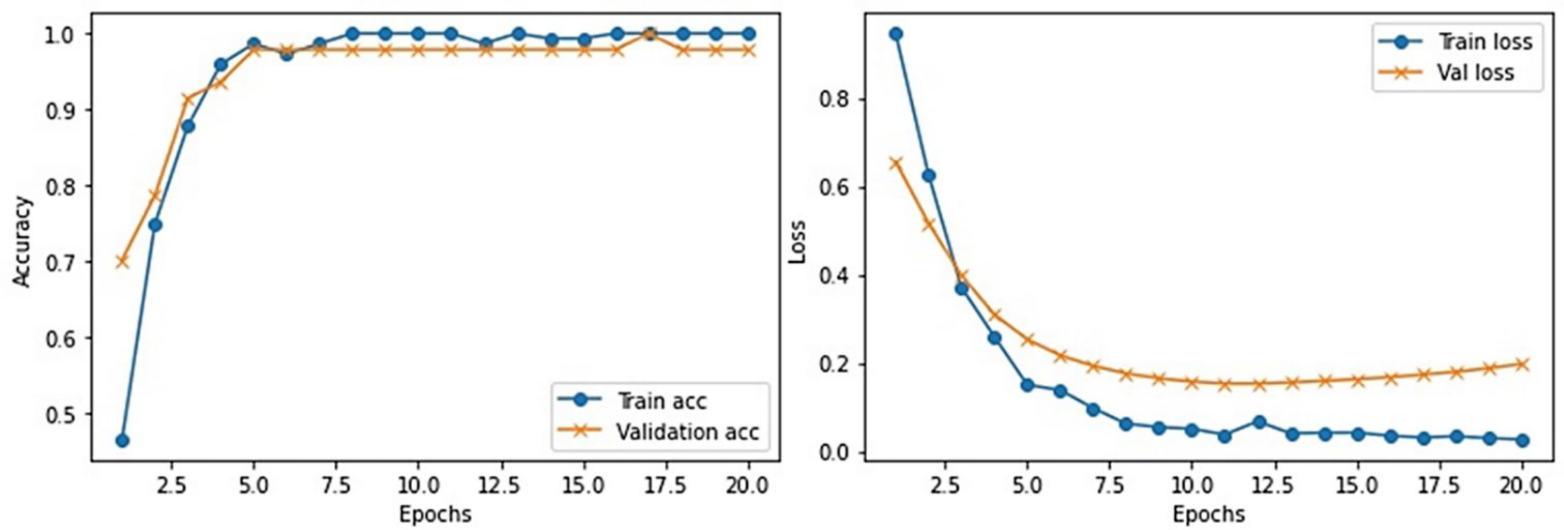
Training and validation performance of our proposed WEENet over medical CXI image dataset.

**Figure 4:**
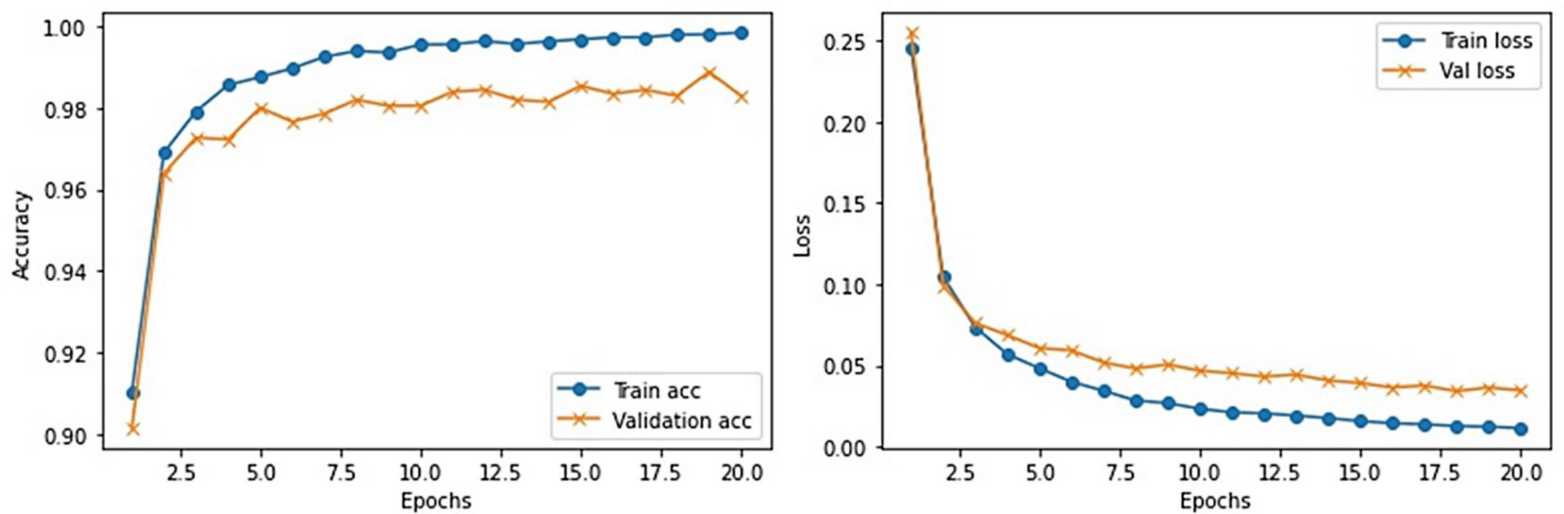
Training and validation performance of our proposed WEENet over medical XDC image dataset.

**Figure 5:**
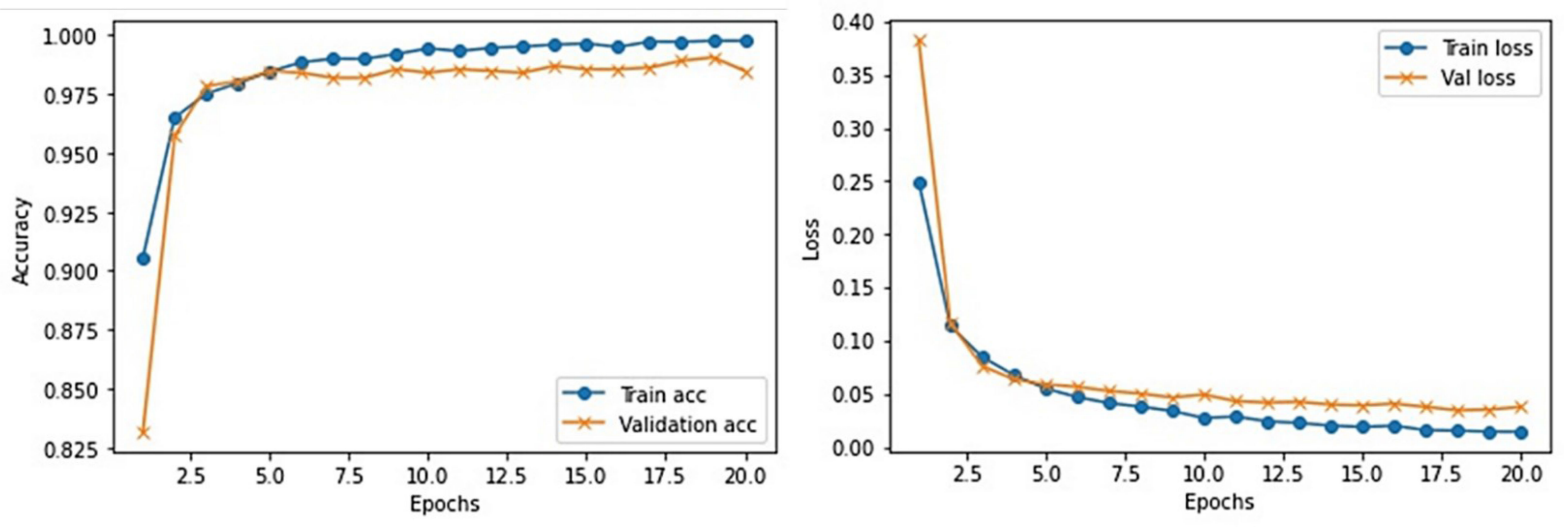
Training and validation performance of our proposed WEENet over medical CRD image dataset.

This section provides the detail of experimental settings and the execution environment used for implementing our proposed WEENet framework. The proposed method is purely implemented in Python (version 3.5) language using Visual Studio Code (VSCode)-integrated development environment. The WEENet concepts are implemented by utilizing a very prominent deep learning framework called Keras on Intel Core i7 CPU equipped with a GPU of Nvidia GTX having 6 GB onboard memory. The proposed WEENet architecture is trained on three different datasets including CXI, XDC, and CRD with the same configuration of hyperparameters, i.e., number of epochs, batch size, learning rate, weight decay, etc. The training and validation performances of our WEENet on CXI, XDC, and CRD datasets are visually depicted in **Figures 3**–**5**, respectively.

### 3.2 Details of the Datasets

For experimental evaluation, we have used three publicly available datasets (18, 32, 33) to validate the performance of our proposed method compared with other SOTA CNN architectures. These datasets contain chest x-ray images of positive and negative COVID-19 patients assigned with corresponding labels, i.e., COVID-19 and normal. The statistical details of the abovementioned datasets are listed in **Table 2**. Besides these datasets, there are several other publicly available datasets commonly used for COVID-19 classification. However, most of them are either imbalance or have weak diversity leading to poor performance. Therefore, we selected CXI, XDC, and CRD datasets from the publicly available listed datasets in **Table 3**. The detail of each dataset is given in **Table 3** including publishing year, name of the dataset, number of samples in COVID class and non-COVID class, methods of evaluation, and experimental outcomes in terms of sensitivity, specificity, and accuracy.

**Table 2:** Number of samples per class in original and augmented datasets.

Dataset	Original dataset		Augmented dataset	
	COVID-19	Normal	COVID-19	Normal
CXI (18)	200	5,000	3,000	3,000
XDC (32)	94	94	940	940
CRD (33)	3,616	10,192	10,848	10,848

**Table 3:** Detailed information of the collected SOTA including techniques and their other important remarks.

Ref.	Year	Dataset	COVID	Non-COVID	Technique	Inclusion	Performance (%)
(18)	2020	CXI	200	5,000	ResNet18, ResNet50, SqueezeNet, and DenseNet121	✔	Sensitivity = 98 ( ± 3)
							Specificity = 90
(19)	2021	SARS-COV-2 CT scan dataset	1,252	1,230	ADECO-CNN	✘	Accuracy = 98.99
(21)	2021	COVID-CS	68,626	75,541	Novel joint classification and segmentation along fine-grained pixel-level labels of opacifications	✘	Sensitivity = 95
							Specificity = 93
(22)	2021	CT scan dataset	349	397	Four models for feature extraction and machine learning classifiers for classification	✘	Accuracy = 87.9
(32)	2021	XDC	94	94	Centralized-VGG16 + data augmentation.	✔	Sensitivity = 95.1
							Specificity = 93.0
					Centralized-ResNet50 + data augmentation.		*Sensitivity = 96.8*
							Specificity = 96.2
(33)	2021	CRD	3,616	10,192	U-Net	✔	Accuracy = 98.21
					Modified U-Net		Accuracy = 98.63
Ours	2021	CXI	200	5,000	WEENet	✔	Sensitivity = 85.0
							**Specificity** = **100**
							**Accuracy** = **99.5**
		XDC	94	94		✔	**Sensitivity** = **100**
							Specificity = 95.7
							Accuracy = 97.8
		CRD	3,616	10,192		✔	Sensitivity = 98.6
							*Specificity = 99.6*
							*Accuracy = 99.3*

#### 3.2.1 CXI Dataset

It is one of the most used datasets for COVID-19 diagnosis in medical image analysis community. This dataset contains a total of 184 COVID-19 infected and more than 5,000 normal chest x-ray images. Clearly, the original CXI (18) dataset has imbalance class samples that significantly affect a model’s performance during training. Considering the chances of overfitting during training, we augmented the dataset and balanced the number of images for both COVID-19 and normal class. The number of per class images for both original and augmented CXI dataset is listed in **Table 2**.

#### 3.2.2 XDC Dataset

This dataset is created by collecting a small number of chest x-ray images of positive and negative COVID-19 patients. Overall, this dataset is very small and cannot be used for training large CNN networks. Also, such a lesser amount of image data often leads to model underfitting where model struggles to learn from the data under observation. To avoid such kind of hurdles during training, we augmented the XDC (32) dataset and increased the number of images from 94 to 940 for both COVID-19 and normal class as given in **Table 2**.

#### 3.2.3 CRD Dataset

The COVID-19 radiography dataset is the large-scale chest x-ray image dataset released in different versions. In the first release, they publicly share 219 COVID-19 infected and 1,341 normal chest x-ray images. In the second release, they increased the number of COVID-19 infected chest x-ray images to 1,200. Following this, in the third release, the number of COVID-19 infected chest x-ray images is increased to 3,616 and normal chest x-ray images to 10,192. In this paper, we used the final release of the CRD (33) dataset, whose statistical details are presented in **Table 2**.

### 3.3 Evaluation Metrics

In image classification problem, the performance of trained CNN model is mostly evaluated by conducting quantitative assessment *via* commonly used classification performance metrics. These metrics can be easily computed with the help of confusion matrix by forwarding the actual class labels and predicted labels. Following this strategy, in this paper, we used eight commonly used performance evaluation metrics that include true positive (TP), false positive (FP), false negative (FN), true negative (TN), sensitivity, specificity, accuracy, and receiver operating characteristics (ROC) for validating the classification performance of our WEENet. The values of TP, FP, FN, and TN are retrieved from the confusion matrix and the sensitivity, specificity, accuracy, and ROC metrics are computed accordingly as Eq. (3) to Eq. (6).


(3) $$\text{Sensitivity}=\frac{\text{TP}}{\text{TP}+\text{FN}}$$

(4) $$\text{Specificity}=\frac{\text{TN}}{\text{TN}+\text{FP}}$$

(5) $$\text{Accuracy}=\frac{\text{TP}+\text{TN}}{\text{TP}+\text{TN}+\text{FP}+\text{FN}}$$

(6) ROC=Sensitivity∼R∼Specificity

Here, sensitivity indicates the number of correctly classified positive samples over the total number of positive samples. Similarly, specificity represents the number of correctly classified negative samples over the total number of negative samples. Accuracy is a generic classification metric that indicates the total number of correct classifications over the total number of samples. Finally, ROC metric represents the relationship (indicated by symbol ~R~) between specificity and sensitivity.

### 3.4 Cross-Corpse Evaluation and Robustness Analysis

**Figure 6:**
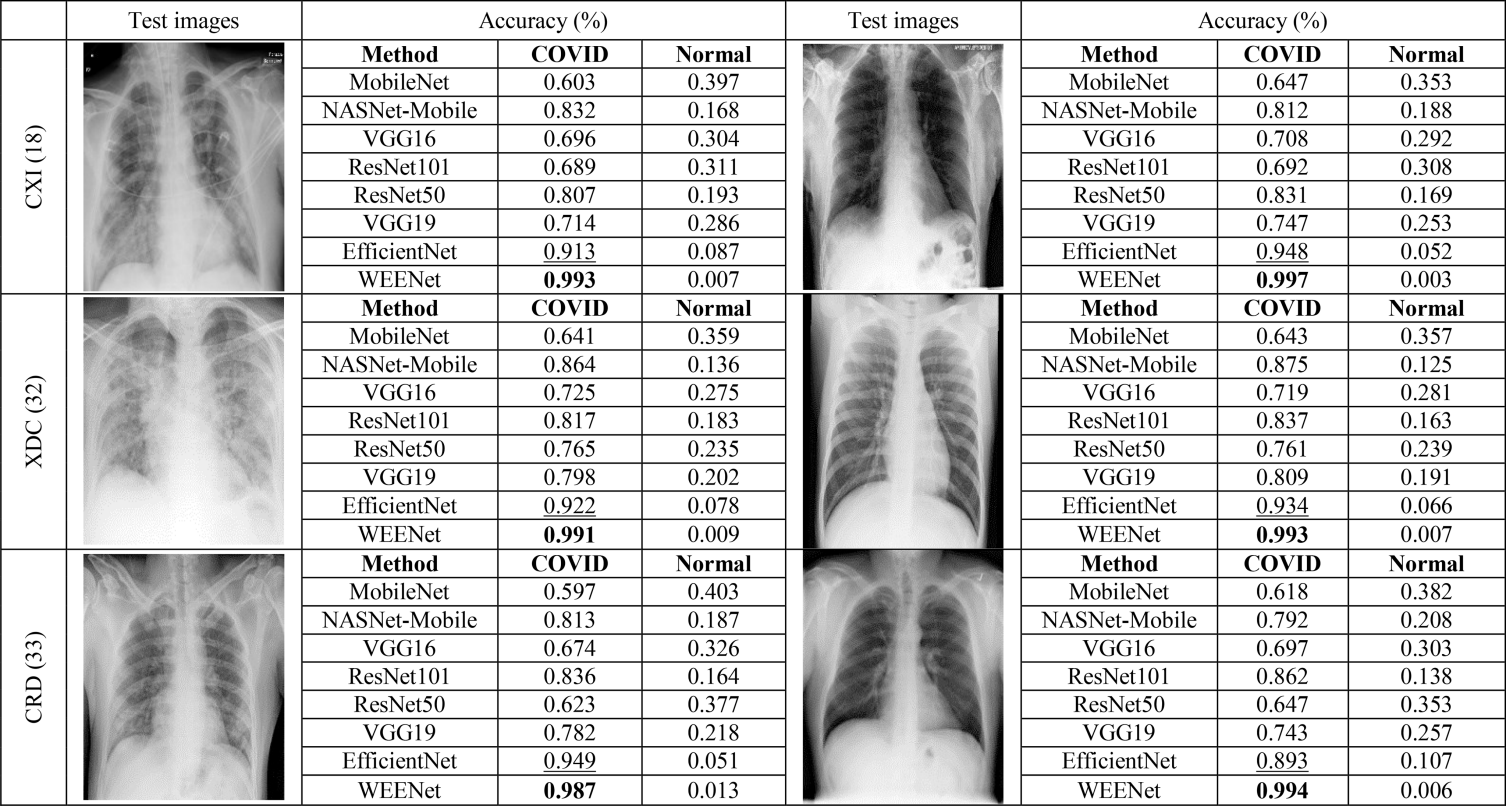
Robustness analysis of our proposed method against other SOTA CNNs on randomly selected test images from each dataset and the corresponding predictions made by each method.

The generalization of a system plays an important role especially when dealing with uncertain computational environment, where data under the observation is semantically different from the data used for training the algorithm. Bearing this in mind, we proposed a new evaluation strategy called cross-corpse evaluation for validating the generalization and robustness of our proposed system in uncertain environment. In this new evaluation strategy, first, we evaluated the performance of our method against other SOTA on test sets of the same datasets used for training. While in the second round of experiments, we assessed the performance of the proposed approach compared with the underlined investigated CNNs on test sets of the datasets other than training datasets, which is termed as cross-corpse evaluation. The obtained quantitative results for both the same dataset and cross-corpse evaluation strategy are presented in **Tables 4**, **5**. It can be easily perceived that the obtained accuracy score for cross-corpse evaluation is comparatively lower than that of the original dataset, yet the accuracy scores indicate the better generalization performance. Furthermore, the reported quantitative results in **Tables 4**, **5** verify the overwhelming performance of our method by obtaining the highest accuracy across each dataset in both the same dataset and cross-corpse evaluation. We also evaluated the qualitative performance of our method against SOTA by doing classification on randomly collected images from the test sets of each experimented dataset. The prediction results for randomly selected images from each experiment dataset are shown in **Figure 6**, where it can be noticed that our method provides the best prediction results compared with other SOTA methods for COVID-19 classification.

**Table 4:** Performance comparison of several deep learning-based models over benchmark datasets.

Dataset name	Model	Original dataset								Cross-corpse evaluation
CXI (18)		TP↑	FP↓	FN↓	TN↑	Sensitivity↑	Specificity↑	Accuracy↑	ROC↑	Accuracy↑
	MobileNet (38)	12	24	485	515	0.024	0.955	0.508	0.424	0.435
	NASNet-Mobile (39)	**26**	**10**	254	746	0.092	0.986	0.745	0.734	0.714
	VGG16 (34)	17	19	380	620	0.042	0.970	0.614	0.546	0.597
	ResNet101 (35)	21	15	402	598	0.049	0.975	0.597	0.591	0.557
	ResNet50 (35)	19	17	341	659	0.052	0.974	0.654	0.593	0.604
	VGG19 (34)	16	20	258	742	0.058	0.973	0.731	0.593	0.710
	EfficientNet (31)	24	12	179	811	0.118	0.985	0.813	0.743	0.847
	**WEENet**	25	11	**97**	**903**	**0.200**	**0.988**	**0.895**	**0.799**	**0.887**
XDC (32)	MobileNet (38)	11	9	8	12	0.578	0.571	0.585	0.575	0.553
	NASNet-Mobile (39)	14	6	4	16	0.777	0.727	0.750	0.750	0.782
	VGG16 (34)	13	7	9	11	0.590	0.611	0.600	0.600	0.574
	ResNet101 (35)	16	4	6	14	0.723	0.777	0.750	0.750	0.745
	ResNet50 (35)	15	5	7	13	0.681	0.722	0.700	0.700	0.617
	VGG19 (34)	12	8	6	14	0.666	0.636	0.650	0.650	0.592
	EfficientNet (31)	18	2	4	16	0.818	0.888	0.850	0.850	0.817
	**WEENet**	**19**	**1**	**2**	**18**	**0.904**	**0.947**	**0.925**	**0.925**	**0.894**
CRD (33)	MobileNet (38)	231	132	466	554	0.331	0.807	0.567	0.590	0.517
	NASNet-Mobile (39)	309	54	274	746	0.530	0.930	0.762	0.791	0.698
	VGG16 (34)	244	119	436	584	0.358	0.830	0.598	0.622	0.578
	ResNet101 (35)	251	112	464	556	0.451	0.833	0.583	0.618	0.514
	ResNet50 (35)	223	140	499	521	0.308	0.788	0.538	0.563	0.482
	VGG19 (34)	187	176	238	782	0.440	0.816	0.700	0.641	0.697
	EfficientNet (31)	**329**	**34**	138	882	0.704	0.962	0.875	0.886	0.837
	**WEENet**	**329**	**34**	**84**	**936**	**0.796**	**0.964**	**0.914**	**0.912**	**0.894**

**Table 5:** Performance comparison of several deep learning-based models over augmented datasets.

Dataset name	Model	Original dataset								Cross-corpse evaluation
CXI (18)		TP↑	FP↓	FN↓	TN↑	Sensitivity↑	Specificity↑	Accuracy↑	ROC↑	Accuracy↑
	MobileNet (38)	219	149	134	266	0.620	0.641	0.631	0.630	0.583
	NASNet-Mobile (39)	307	61	64	336	0.827	0.846	0.837	0.837	0.784
	VGG16 (34)	280	88	126	388	0.689	0.815	0.757	0.758	0.693
	ResNet101 (35)	249	119	101	299	0.711	0.715	0.713	0.712	0.647
	ResNet50 (35)	307	61	98	302	0.758	0.832	0.793	0.795	0.691
	VGG19 (34)	291	77	79	321	0.786	0.806	0.796	0.797	0.738
	EfficientNet (31)	339	29	37	363	0.901	0.926	0.914	0.914	0.883
	**WEENet**	**364**	**4**	**3**	**397**	**0.991**	**0.990**	**0.991**	**0.991**	**0.947**
XDC (32)	MobileNet (38)	61	33	39	55	0.610	0.625	0.617	0.617	0.587
	NASNet-Mobile (39)	83	11	19	75	0.813	0.873	0.840	0.840	0.816
	VGG16 (34)	71	23	30	64	0.703	0.735	0.718	0.718	0.687
	ResNet101 (35)	77	17	20	74	0.793	0.813	0.803	0.803	0.774
	ResNet50 (35)	72	22	19	75	0.791	0.773	0.781	0.782	0.698
	VGG19 (34)	68	26	20	74	0.772	0.740	0.755	0.755	0.714
	EfficientNet (31)	88	6	12	82	0.880	0.931	0.904	0.904	0.817
	**WEENet**	**93**	**1**	**2**	**92**	**0.979**	**0.989**	**0.984**	**0.984**	**0.974**
CRD (33)	MobileNet (38)	759	261	402	618	0.653	0.703	0.675	0.675	0.597
	NASNet-Mobile (39)	822	198	174	846	0.825	0.810	0.817	0.818	0.798
	VGG16 (34)	784	236	433	587	0.644	0.713	0.672	0.672	0.586
	ResNet101 (35)	833	187	116	904	0.877	0.828	0.851	0.851	0.880
	ResNet50 (35)	751	269	478	542	0.611	0.668	0.633	0.634	0.595
	VGG19 (34)	798	222	198	822	0.801	0.787	0.794	0.794	0.742
	EfficientNet (31)	947	73	103	917	0.901	0.926	0.913	0.914	0.847
	**WEENet**	**1004**	**16**	**19**	**1001**	**0.981**	**0.984**	**0.982**	**0.983**	**0.914**

### 3.5 Comparison With Other CNN Models for COVID-19 Classification

**Figure 7:**
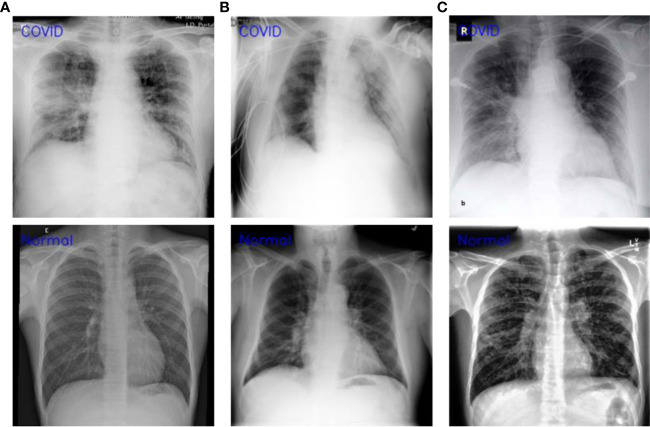
Visual results of WEENet over each dataset **(A)** CXI, **(B)** XDC, and **(C)** CRD datasets.

This section presents the comparative analysis of our proposed WEENet with other SOTA methods for COVID-19 classification on test sets of CXI, XDC, and CRD datasets. For comparative analysis, we evaluated the performance of our method and compared it with SOTA including MobileNet (38), NASNet-Mobile (39), VGG16 (34), ResNet101 (35), RestNet50 (35), VGG19 (34), and EfficientNet (31). To investigate the performance of our method and validate the effectiveness of the proposed data augmentation strategy, we conducted experiments on both original datasets and augmented datasets and compared the results with the SOTA methods. The obtained results for the original dataset are given in **Table 4**, where it can be perceived that our proposed WEENet outperforms all comparative CNNs on original datasets across each evaluation metric except NASNet-Mobile (39) that performs comparatively better than our method in terms of TP and FP on the CXI dataset. On the other hand, the obtained results on augmented datasets are given in **Table 5**, where it can be noticed that our proposed WEENet achieved the best results by overwhelming the SOTA CNNs across each evaluation metric, thus showing its superiority and efficiency for COVID-19 classification in medical chest x-ray images. We also compared our WEENet architecture with other SOTA CNN-based COVID-19 classification approaches and reported the results in **Table 6**. The reported results reflect the dominancy of our WEENet on CRD dataset across each evaluation metric Although our method obtained comparatively lower values for sensitivity and specificity on the CXI and XDC datasets, still our method attained best results on the same datasets across the other two evaluation metrics. The best reported results presented in **Tables 3**–**6** are highlighted in the bold text while the runner up scores are indicated in the underlined text. Furthermore, some visual results of the proposed WEENet over test set of each dataset are given in **Figure 7**.

**Table 6:** Performance comparison of the proposed WEENet with other baseline models.

Dataset	Model	Original dataset		
CXI (18)		Sensitivity↑	Specificity↑	Accuracy↑
	SqueezeNet (40)	**0.980**	0.920	–
	WEENet	0.850	**1.0**	**0.995**
XDC (32)	ResNet50 (35)	0.981	**0.958**	0.970
	WEENet	**1.0**	0.957	**0.978**
CRD (33)	ChestNet (41)	0.962	0.972	0.962
	WEENet	**0.986**	**0.996**	**0.993**

### 3.6 Feasibility Analysis for 5G-Enabled IoMT Environment

**Figure 8:**
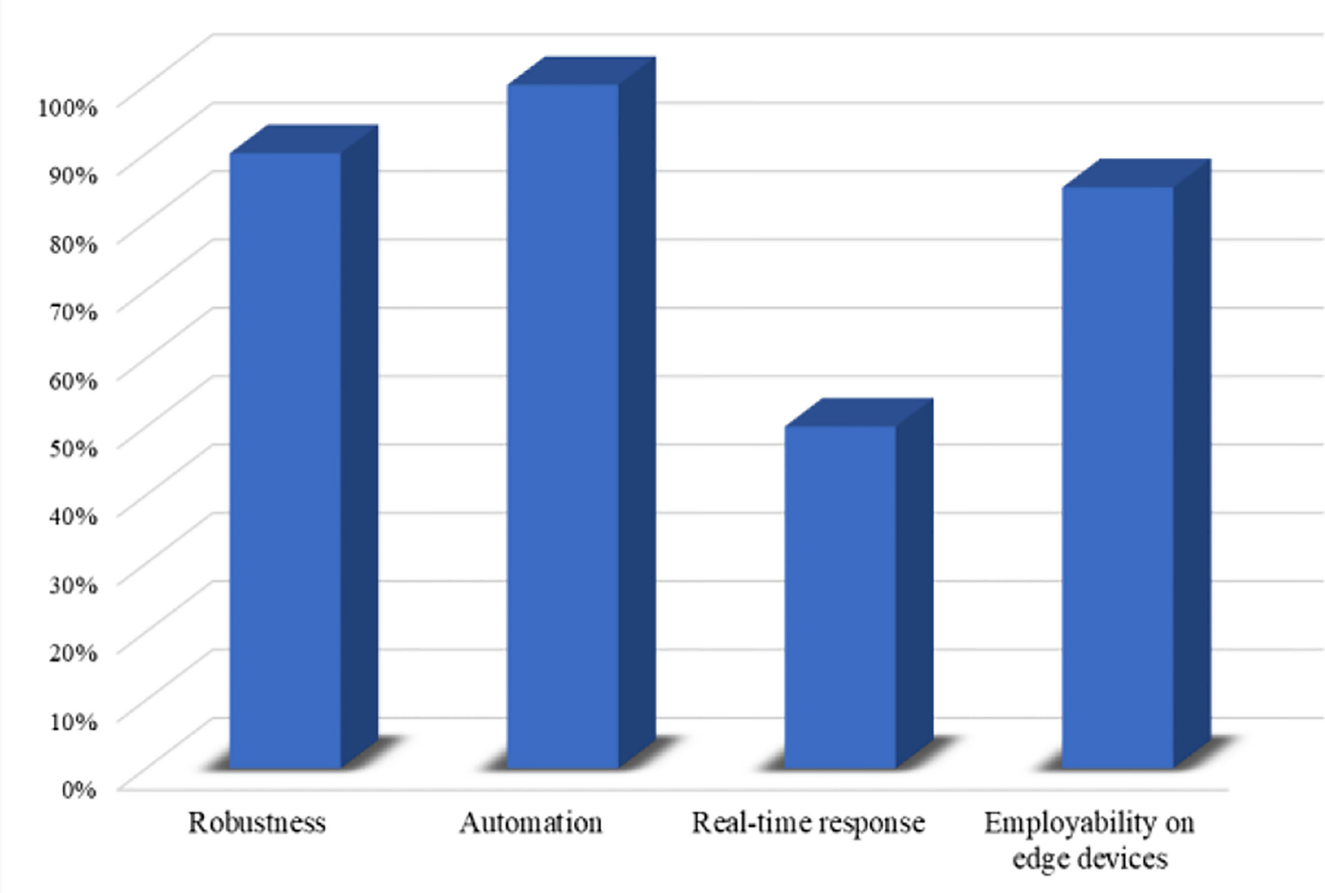
Feasibility assessment of our proposed WEENet for 5G-enabled IoMT environment.

Considering the requirements of 5G-enabled IoMT environment for rapid and accurate smart healthcare systems (42–44), it is essential to analyze the feasibility of a system before deploying in the real world. The feasibility assessment protocols involved different steps to investigate the suitability of a given system for the problem under observation in various aspects such as the robustness of decision-making system, automation, real-time response, and employability on edge-computing platforms. Having this in mind, we conducted feasibility analysis experiments and investigated our proposed WEENet in the abovementioned aspects. Based on the obtained quantitative results in the previous section, we estimated the robustness of our WEENet by averaging the attained accuracy score across all datasets and achieved an average of 90% accuracy. Next, the proposed method meets automation requirements thereby providing fully end-to-end deep learning system. Although, our method takes relatively more time for diagnosing COVID-19 in chest x-ray image, it has limited memory storage requirements for deployment on edge devices, making it a suitable approach for early COVID-19 detection in 5G-enabled IoMT environments. The conducted feasibility assessment findings are depicted in **Figure 8**.

### 3.7 WEENet for Lung Cancer Detection

**Figure 9:**
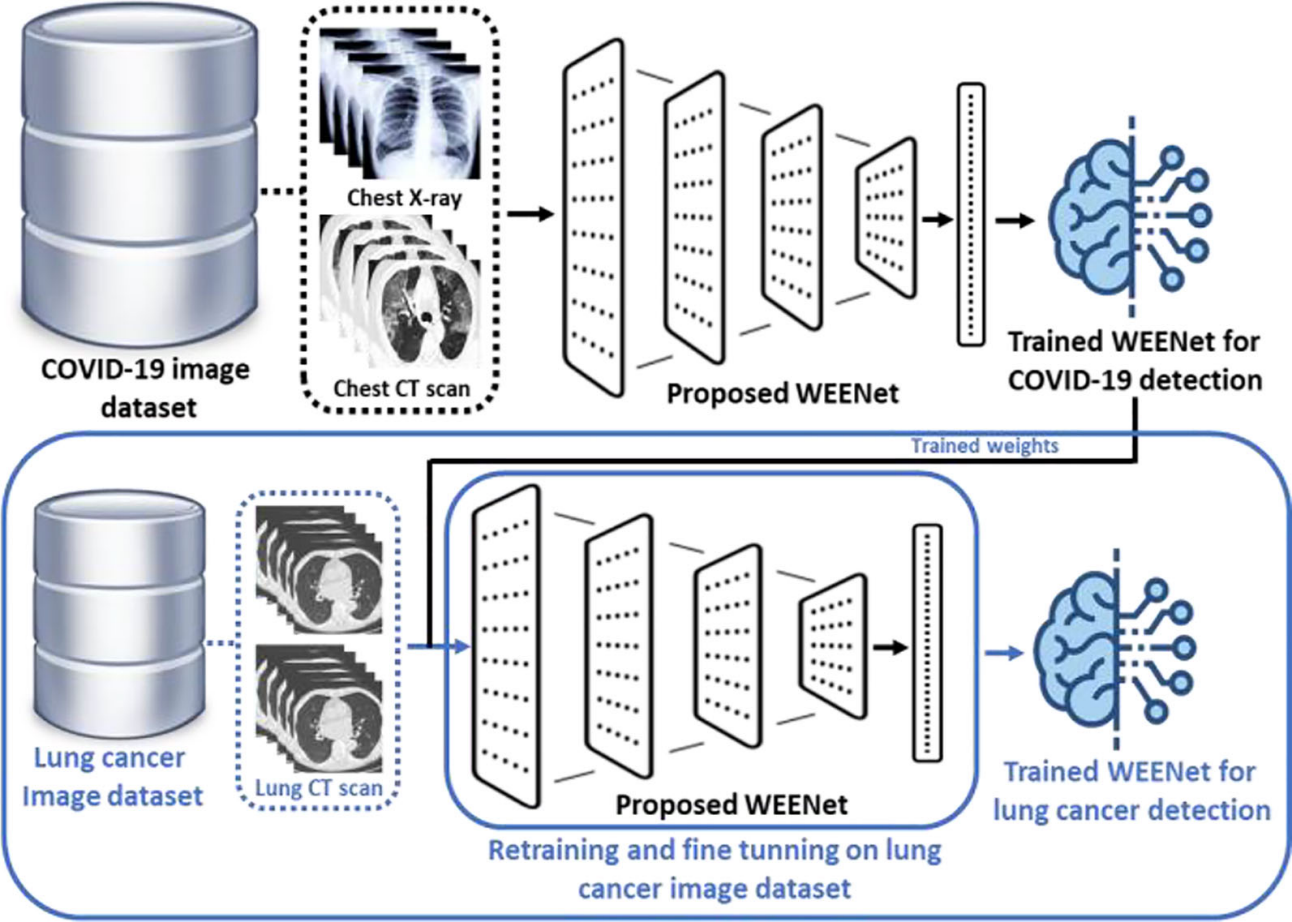
The graphical overview of the reusability process of our proposed WEENet for lung cancer detection task.

In this section, we discuss the effectiveness and reusability of our proposed WEENet framework for early detection of lung cancer in chest CT scan images of infected patients. The deep learning-based early detection of lung cancer (45) can greatly facilitate the doctors and other medical-related individuals to eliminate the cancer cell at first place by providing proper care and treatment to the infected patients. Considering the relevancy in the image data (chest CT scan images) used for COVID-19 detection and lung cancer CT scan images (46), the proposed WEENet framework can be used for lung cancer detection by fine-tunning the architecture on lung cancer image data using transfer learning strategy (47). For efficient retraining of the WEENet architecture, the trained weights (already learned knowledge during training on COVID-19 image data) can be used while training the proposed WEENet on lung cancer image data. The utilization of trained weights will not only reduce the training efforts (in terms of training time) but can also improve the performance of retrained architecture for lung cancer detection. The reusability workflow of our proposed WEENet for lung cancer detection task is depicted in **Figure 9**.

## 4 Conclusion and Future Work

The COVID-19 pandemic started in 2019 and has severely affected human life and the world economy for which different actions are initiated to stop its spread and efficiently handle the pandemic. Such actions include the concept of smart lockdown, development of new devices for temperature checking, early detection of COVID-19 using medical imaging techniques, and treatment plans for patients with different risk levels. This work supports the necessary action of early COVID-19 detection using medical chest x-ray images in 5G-enabled IoMT environment, contributing to the management of COVID-19 pandemic. Considering the limited available medical imaging data and different conflicting performance metrics for early COVID-19 detection, in this work, we investigated deep learning-based frameworks for effective modeling and early diagnosis of COVID-19 from medical chest x-ray images in IoMT-enabled environment. We proposed “WEENet” for COVID-19 diagnosis using efficient CNN architecture and evaluated its performance on three benchmark medical chest x-ray and CT image datasets using eight different evaluation metrics such as accuracy, ROC, robustness, specificity, and sensitivity etc. We also tested the performance of our method using cross-corpse evaluation strategy. Our results are encouraging against SOTA methods and will support healthcare authorities in analyzing medical chest x-ray images of infected patients and will assist the management of the COVID-19 pandemic in IoMT environments.

The reported results are better than SOTA methods, but model size is not the best among all methods under consideration (though better than majority of the models). This is due to some of the architectural layers, tuned to balance the performance metrics towards optimization. More investigation is needed to further reduce the model size without affecting the performance, which is one of our plans. We also plan to extend this work to a multiclass problem including mild, moderate, and severe as discussed in COVIDGR dataset (48) from the University of Granada, Spain.

## Data Availability Statement

The original contributions presented in the study are included in the article/supplementary material. Further inquiries can be directed to the corresponding authors.

## Author Contributions

KM, HU, and ZK contributed to the idea conceptualization, data acquisition, implementation, experimental assessment, manuscript writing, and revision. AS contributed to the data acquisition and manuscript revision. AA, MA, MK, MHAH contributed to acquisition and interpretation of data. KMM, MH, and MS contributed to the design of study and revision of manuscript.

## Funding

This work was supported by the Deputyship for Research & Innovation, Ministry of Education in Saudi Arabia for funding this research work through the project number 959.

## Conflict of Interest

The authors declare that the research was conducted in the absence of any commercial or financial relationships that could be construed as a potential conflict of interest.

## Publisher’s Note

All claims expressed in this article are solely those of the authors and do not necessarily represent those of their affiliated organizations, or those of the publisher, the editors and the reviewers. Any product that may be evaluated in this article, or claim that may be made by its manufacturer, is not guaranteed or endorsed by the publisher.
